# “Nobody Seems to Know Where to Even Turn To”: Barriers in Accessing and Utilising Dementia Care Services in England and The Netherlands

**DOI:** 10.3390/ijerph182212233

**Published:** 2021-11-22

**Authors:** Clarissa Giebel, Sarah Robertson, Audrey Beaulen, Sandra Zwakhalen, Dawn Allen, Hilde Verbeek

**Affiliations:** 1 Institute of Population Health Sciences, University of Liverpool, Liverpool L69 3GL, UK; 2 NIHR ARC NWC, Liverpool L69 3GL, UK; dawnallenhenna@gmail.com; 3 Alderhey NHS Foundation Trust, Liverpool L14 5AB, UK; sarah.robertson@alderhey.nhs.uk; 4 Department of Health Services Research, Care and Public Health Research Institute, Maastricht University, 6200 MD Maastricht, The Netherlands; a.beaulen@maastrichtuniversity.nl (A.B.); s.zwakhalen@maastrichtuniversity.nl (S.Z.); h.verbeek@maastrichtuniversity.nl (H.V.)

**Keywords:** dementia, health inequalities, social support services, social care

## Abstract

Background: Accessing post-diagnostic care can be difficult for people with dementia and their informal carers. Little is known, however, about the determinants of barriers to access, and how these might vary between countries. The aim of this study was to explore potential inequalities in access to formal dementia care services between England and the Netherlands, specifically from more disadvantaged areas. Methods: This was a mixed-methods study, involving semi-structured qualitative interviews and a carer questionnaire. People with dementia and informal carers were recruited by clinicians. The postal survey was co-produced with people with dementia, informal carers, and health care professionals. The survey asked carers about their own and their relatives with dementia’s, social support service usage and financing; as well as how they were made aware of services and whether they required more support. Qualitative transcripts were analysed by two researchers in each country using thematic analysis. Results: A total of 103 carer questionnaires were received by post and 13 interviews were conducted with people with dementia and family carers between January 2020 and April 2020. Many services were accessed via self-funding. Thematic analysis generated five core themes: Health literacy; Having faith and lack of faith; Service suitability; Structural issues surrounding service provision; and Financing care. One major difference between both country’s systems of care were the case manager and network support which people with dementia and carers benefitted from in the Netherlands, which was rarely the case in the UK. Conclusions: People with dementia and informal carers need to be supported better in accessing formal dementia care services in both the UK and the Netherlands, whilst some learning can be taken to improve access.

## 1. Introduction

Dementia affects an estimated 55 million people worldwide [1]. Specifically, there are an estimated 920,000 people living with dementia in the UK [2], with an estimated 280,000 people living in the Netherlands [3]. Depending on the dementia subtype, people with dementia (PwD) experience various difficulties and needs, including cognition and language [4], everyday functioning problems [5], behavioural problems [6], and mobility limitations [7].

People affected by dementia benefit from appropriate post-diagnostic support, which can include anything from receiving a paid carer coming to the home, accessing support groups and activities in the community, information, a befriending service, visiting day care centres, psychological therapy, to getting equipment adaptations to the home, or accessing a care home, which can be very costly, increasing in cost by the level of dependence the person with dementia experiences [8,9]. Of numerous different types of formal dementia care, personal and domestic home care, as well as access to day care centres has been highlighted by family carers and care professionals to be of primary importance [10]. Additionally, family carers are also eligible to receive adequate support, for example by accessing a carer support group or getting respite care. However, evidence indicates that post-diagnostic support in dementia is often inadequate and fragmented, both for people with dementia and for their family carers, by not offering the type of support needed for individuals or being too difficult to access [11,12,13,14]. Considering the large costs associated with dementia [2], it is not surprising that family carers provide a large amount of informal care, estimated to equate to £13.9 billion a year in the UK alone [2]. Improving post-diagnostic support and providing better coverage however is associated with improved independence in dementia [15]. Therefore, it is important to understand the reasons behind the fragmented service provision for better coverage to be provided.

People with dementia, and their informal carers, can experience a myriad of different inequalities in accessing the formal care they or their relative needs. Whilst research into dementia care inequalities is receiving more attention [16,17,18], there are still many gaps that need to be addressed. Living in a rural setting for example is linked to reduced dementia service uptake [19,20,21]. This is because of the limited service provision in rural areas, and the long time it takes to use public or private transport. Other evidence has indicated that being from a more affluent background increases the likelihood of getting access to anti-dementia drugs by 25% in the UK [22]. In a European study, Lethin et al. [23] showed, for example, that formal support for informal dementia carers was available yet under-utilised, which to some degree supports more recent evidence into formal dementia care in a deprived region in the North West of England [12]. The recent study also showed how formal care services were under-utilised and not well accessed, but often there was no availability or funding support in particular postcodes. To existing knowledge, there is no Dutch-specific investigation of inequities in accessing dementia care, except for part of some emerging European-wide comparisons. Thus, with a lack of research exploring multiple causes of inequalities in dementia care [24], especially across different countries, more research is required to compare access to and potential barriers to utilisation of formal dementia care across different settings.

The COVID-19 pandemic has brought on new inequalities and difficulties in utilising dementia care, whilst exacerbating existing ones also [12,25,26]. Generally, the pandemic has had a tremendously negative impact on the lives of people living with dementia and informal carers, illustrated by difficulties in accessing social support services and the emotional impact of experiencing lockdown [27,28]. Carers have noticed their relatives with dementia to deteriorate faster, for example [29], which has also been supported by findings on the cognitive severity in people living with dementia [30].

By focusing on health inequalities in dementia care, this study clearly addresses the overall recommendation of the Alzheimer’s Society’s roadmap to advance dementia research and care by 2025, with all Goals suggested to address inequalities to enable equitable access to care [31]. Improved knowledge of the experiences of people with dementia and their informal carers on potential barriers to accessing dementia care services can help to develop strategies to remove these barriers to enable anyone from any socio-economic background to access the right care at the right time. This study therefore had two aims: First, to document usage of dementia-related social support services and potential inequalities in usage both in England and the Netherlands using a survey; and second, to explore the experiences of informal carers and people living with dementia in using these services in-depth via qualitative interviews.

## 2. Methods

### 2.1. Design

We conducted a cross-sectional international study using a mixed-methods approach. Given limited understanding of the barriers in accessing formal dementia care, we sought to explore the scale of the issue using a questionnaire, whilst exploring the reasons behind difficulties in access via semi-structured interviews. Both components—the survey and the interviews—were conducted simultaneously, and allowed a richer understanding of the situation then would be possible by only utilizing one methodological approach.

### 2.2. Participants and Recruitment

People aged 18+ with a diagnosis of any type of dementia and informal carers were eligible to take part in this study. The diagnosis of dementia was confirmed by the healthcare professional where possible. Participants had to live either in the North West Coast area of England or in the province of Limburg, in the Netherlands. Both areas are some of the most deprived regions in both countries, with residents in Limburg, in the South of the Netherlands, having a lower life expectancy and higher disease burden (Department for Communities and Local Government, 2015 CBS/RIVM). In England, participants were recruited via leaflets provided by memory clinic staff and general practitioners, as well as by care home staff, and recruitment was conducted across six NHS Trusts. In addition, participants were recruited via the National Institute for Health Research’s ENRICH network, which is a network of research-supportive care homes, as well as via Join Dementia Research, a network of registered people with dementia, carers, and healthy volunteers who are interested in taking part in research. In the Netherlands, carers were recruited via similar sources via support groups and care homes. Staff handed out the questionnaire and freepost return envelope to family carers, or sent out the questionnaire to the carers directly.

Ethical approval in England was obtained from the North West Haydock Research Ethics Committee (Ref: 19/NW/0320) and in the Netherlands from the Medisch Ethische Toetsingscommissie Zuyderland en Zuyd Hogeschool (Ref: METCZ20190089) prior to study commencement. Informed consent was obtained differently for the survey and the interviews. For the survey, participants signed the consent form and returned this in a pre-paid envelope. For the interviews, pre-pandemic, participants signed the consent form at the beginning of the face-to-face interview, whilst since COVID participants were posted the consent form or emailed, then signed and either posted these or took a photo and sent this via email.

### 2.3. Data Collection and Variables

All data were collected between October 2019 and April 2020. Data collection of the survey had to be stopped as it was considered unsafe and logistically not possible to post out the surveys any longer due to the COVID-19 pandemic, with recruiting NHS Trusts having put non-COVID-19 research and care on hold. Before the pandemic, questionnaires were posted and handed out to informal carers of people with dementia alongside an information sheet, consent form, and freepost return envelope, in which they could return the survey and signed consent form.

#### 2.3.1. Questionnaire

The questionnaire was developed via a consultation meeting with healthcare professionals, including psychologists, which helped identify the various types of dementia care services, amongst others. Subsequently, the draft questionnaire was further developed and edited with three public advisers (two carers of people living with dementia and one person living with dementia). The questionnaire comprised questions on basic demographics of the family carer (age, gender, ethnicity, relationship to person with dementia, and years of education) and of the person with dementia they cared for (age, gender, ethnicity, dementia diagnosis, length since dementia diagnosis, living situation, postcode, years of education, and last job before retirement). Questions also asked about the hours of informal care provided each week, and the time of diagnosis and point in time when symptoms were first apparent. Where carers were caring for a person with young-onset dementia (YOD) (diagnosed before the age of 65), they were asked about accessing YOD specific services and to what extent they prefer YOD-specific services.

Another set of questions in the questionnaire concerned the access to and funding for various different types of formal dementia services, including paid carers, support groups, clinical support, day care centres, and care homes, and whether any difficulties, and which (i.e., availability of services, lack of financing, distance to services, and time of day), were encountered when trying to access formal dementia care services. Additionally, carers were asked how they found out about the services, whether they need more support, and which type of health care service the PwD has accessed in the past 12 months (doctor, planned or unplanned hospital admission, and other).

#### 2.3.2. Interviews

Similarly, recruitment for interviews was equally affected, by being able to conduct one previously booked in interview over the phone after the pandemic outbreak, yet no further recruitment was possible. Thus, the majority of interviews were conducted in the home of the person with dementia/carer, at the University, or in a quiet room in a care home where the person with dementia was residing, and lasted a maximum of 45 min. After written or verbal informed consent was obtained from the participant(s), and the mental capacity of the person with dementia was assessed prior, the researcher asked both the person with dementia and their informal carer questions about the types of services they have accessed in the past or are currently using; who pays for the services; whether there are any barriers to accessing support services; and how they decided to use specific services, and not others. The interview guide is attached in Appendix A.

### 2.4. Data Analysis

#### 2.4.1. Quantitative Data

Demographic characteristics and service usage were analysed using frequency analysis in SPSS 25. Bivariate correlation analysis was used to explore the relationship between carers’ education and service usage.

#### 2.4.2. Qualitative Data

Data were coded for themes according to thematic analysis [32] by two members of the research team in each country (CG, SR; HV, AB), specifically using Braun & Clarke’s different phases of thematic analysis. Coders are dementia care researchers and have in-depth experience in analysing qualitative interview data. Both researchers familiarized themselves with the data and transcripts (Phase 1), analysed the data individually and generated codes and searched for themes (Phase 2 and 3), and then discussed and compared these. This involved reviewing the themes jointly and defining and naming them (Phase 4 and 5). Where only one researcher generated a theme, this was discussed to establish whether it could be merged with a different theme or whether it should stand on its own.

### 2.5. Public Involvement

One person living with Lewy Body dementia, one former family carer, and one former care home staff member were involved in designing the study from the beginning. They provided feedback on study documents, attended some of the English interviews alongside the lead investigator (CG), and helped interpret the data. In addition, all helped in the dissemination of the findings, by reviewing this manuscript and by drafting jointly a two-page lay summary for the general public.

## 3. Results

A total of 103 informal carers (89 in England; 14 in the Netherlands) took part in the survey, with a total of 13 interviews conducted in both countries (7 in England, 6 in the Netherlands). PwD were on average 78 (±8) years old [Range 51–94], female (*n* = 74, 71.8%), and lived with a diagnosis of Alzheimer’s Disease dementia (*n* = 58, 57.4%), followed by vascular dementia (*n* = 21, 20.8%) and mixed dementia (*n* = 14, 13.9%). Family carers were on average 67 (±10) years old [Range 42–88], female (*n* = 54; 52.4%), and were mostly spouses (*n* = 52, 59.1%) and adult children (*n* = 31, 35.2%). Table 1 shows the demographic characteristics of survey participants and their relatives with dementia.

### 3.1. Survey on Social Support Service Usage

**Figure 1:**
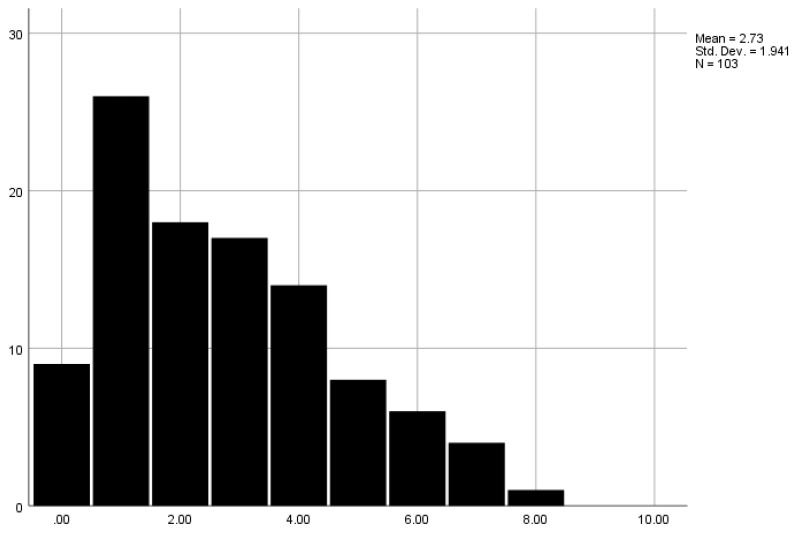
Number of services utilised. Legend. X-axis shows the number of combined services a participant was accessing. Y-axis shows the number of people who accessed a given number of services.

**Figure 2:**
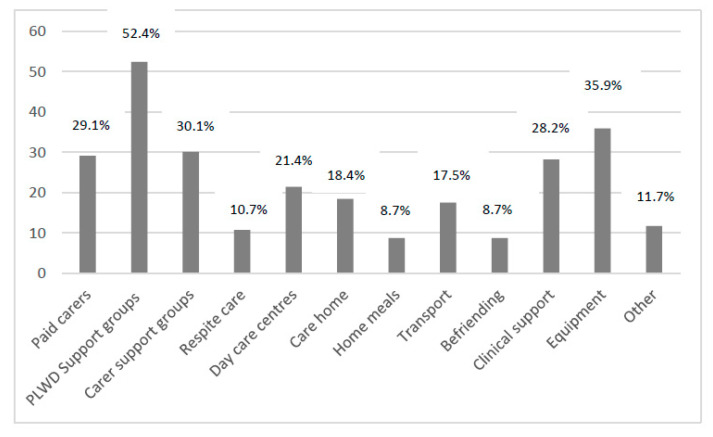
Social support service usage. Legend. PLWD = Person living with dementia.

Overall, 8.7% did not access any services, with one service being accessed by the largest proportion of carers (*n* = 26, 25.2%). One carer accessed eight services (1%). Figure 1 highlights the number of carers who accessed different numbers of services. Specifically, support groups for people living with dementia were accessed by more than half of participants (52.4%), with equipment (35.9%), carer support groups (30.1%), paid home carers (29.1%), and clinical support (such as post-diagnostic support groups specifically provided by NHS services right after a diagnosis) (28.1%) accessed by a third of carers. Home meals and befriending services were accessed the least (8.7% each) (see Figure 2 for further details on service usage). Across those services, paid home care, care homes, and home meals were mostly self-funded. Table 2 details the proportion of carers having accessed different services and their proportion of being fully and partially self-funded.

Bivariate correlation analysis revealed no significant correlation between carers’ years of education and service usage (yes/no) (*p* = 0.820) and number of services used (*p* = 0.817).

### 3.2. Interviews

Across the 13 cross-country interviews (7 in England, 6 in the Netherlands), five themes emerged which addressed a mix of structural and personal barriers to accessing and utilizing post-diagnostic dementia care: Health literacy; Accepting help; Service suitability; Structural service barriers and enablers; and Financing care. Table 3 illustrates the themes and sub-themes further.

**THEME 1: Health literacy**

**Knowledge and communication skills**

Being able to communicate one’s needs and seeking out information about services and support available was both a personal and structural facilitator to accessing care. Information about the condition and services seemed to mostly be provided early, or shortly after the diagnosis, without any information throughout the condition which is adapted to the changing needs of those living with dementia and informal carers. Especially in the UK, informal carers were thus complaining about how they received very little to no information about services in the UK. This is a structural barrier to accessing care, whilst the personal barrier relates to carers’ own skills in seeking out information. Carers recognised that they were lucky in knowing how to find out information, and how they would be lost without these skills.

*“I don’t know what’s available you see. No one’s ever told me.” UK ID02, Person with dementia*

*“I suppose I am lucky because I am younger and I know how to manage these things. (…) I always thought, as I also told my children: ‘Those poor people who don’t know how to do this.”Dutch Female, spouse, age 77*

**A need to be proactive**

In light of the limited information provided about available services, many carers described their own proactiveness in seeking out help. Carers need to be proactive and search for information themselves to get access to care services. This included people with dementia having to be proactive at times and go so far as to actively seek out a diagnostic assessment, as this would not be provided otherwise. This need to be proactive is thus a personal facilitator to accessing care.

*“I searched around for alternatives looked at, I think I looked everywhere from Crewe to Colwyn Bay but looking around Chester and as soon as I saw the place in Ellesmere Port I thought this is it.”*

*UK ID01, Male carer, son*

*“I myself thought there was something wrong, so I asked for a brain scan. Then I asked: ‘I want a second brain scan.’ However, the doctor didn’t want to arrange this. The neurologist didn’t want to do it. Eventually they made a second brain scan. However, in my hospital they are not specialized in my condition, and that is why I want to go to Rotterdam. I want them to take another look at this brain scan, and compare this one with the one from two years ago, to see whether there is a difference.” Dutch Male, person with dementia, age 54.*

**THEME 2: Accepting help**

Accepting help from services and having, or lacking, trust in the professionals providing the care emerged as one barrier, or facilitator, in accessing care. It is important for carers and people living with dementia to accept help and support, and people living with dementia and carers expressed less faith when they experienced negative health care experiences and had to arrange health care themselves. This was the case in most people interviewed, with carers having to be proactive to find help.

*“I would say the big thing with finding services is trying to negotiate your way through the system and the system wants to keep you at arm’s length.” UK Male carer, son, ID01*.

One Dutch carer was specific about how she accepted all the support that was offered. At times, people may want to accept services but do not feel these adequately provide the support they want.

*“I accepted all help that was offered to me. I have let them come over and I got into business with them. And I think you should not try to be too difficult. And just accept that you need help. That is… yes, I suppose that is 98% of the whole thing.” Dutch Female, spouse, age 67*.

**THEME 3: Service suitability**

Many carers expressed concern over the suitability of services for their relatives with dementia. People with dementia experience different symptoms and have different needs depending on their subtype. This was particularly pronounced in people living with young-onset dementia as opposed to late-onset dementia, with most services only suitable to the interests of those with late-onset dementia. This can also be particularly the case for people with rarer subtypes of dementia, such as Lewy Body or behavioural-variant fronto-temporal dementia, who experience often different needs and symptoms to those living with Alzheimer’s disease dementia. Services may also not be suitable due to the activities on offer and some people less inclined to attend group settings as opposed to face-to-face one-on-one support.

*“what we did was we amended it to suit ourselves because the [Charity] set it up at a time when nobody liked going. It was right in the middle of lunchtime/erm a time when people didn’t want to be there it was like something like 11 til 11 til 1 or something stupid like I cant even remember the time now but it was at a time nobody ever liked going.” UK Female carer, spouse, ID08*.

This lack of person-centredness of services was a structural barrier in utilising services, as they may be available and accessible, yet were not utilised because of these reasons. For some people with dementia, what was on offer was of no interest. Some people may not enjoy going to coffee mornings with peers with dementia, for example. However, it is also feasible that some people with dementia generally do not wish to have any support, regardless of the type on offer. Carers expressed that these services would be of no benefit to their relative, and thus they were not or no longer utilising the service. Often, these can be the only services offered though, which can thus create a vacuum of support and leave people living with dementia without suitable care.

*“The only thing he doesn’t want to do and the only thing he has been offered is to go to these coffee mornings. He doesn’t want it and I’m not forcing him to go.” UK ID02, female carer, wife*.

*“if we went to the lounge after a while my Mum would find it too much, she’d sort of tug on my elbow which is always a sign so things like memory café’s wouldn’t have helped her at all.” UK ID01, male carer, son*.

*“I would like to have an alternative doctor who knows something about it and who could help me in a way that is normally not possible in the hospital. It’s just that I haven’t been able to find an alternative doctor who knows anything about it or can help me.” Dutch male person with dementia, age 54*.

**THEME 4: Structural service barriers and enablers**

**Having one link person**

Accessing dementia care in both the UK and the Netherlands seemed to be facilitated greatly by having a link person to connect with services, thus highlighting a structural barrier to accessing care. In the Netherlands, this person was called a care navigator, and where carers and people living with dementia had a good care navigator, they experienced better access to services. This was reflected in the UK, with Admiral Nurses and occasionally social workers. However, these link persons are rare in the UK and not provided to everyone equally, creating a structural service barrier in accessing care.

*“Because you suddenly enter a world you don’t know anything about. She was very healthy. And suddenly… How am I supposed to…? And then they came, and that was a reassurance. ‘I will do that for you’. That lady, that case manager, she came to meet us and said: ‘I will take care of that.’” Dutch Male carer, spouse, age 79*.

*“I think we just had 1 appointment [with an Admiral Nurse] which is great I still remember it fondly.” UK ID01, male carer, son*

*“the consultant give us a phone number for a social worker and so from February to June the social worker came around and in June things were put in place for us. They told us all about the place we could go to in St Helens, the Veterans support group with dementia, lots of different things that Frank could access but we didn’t know any of all of this.” UK ID04, female carer, spouse*.

Having an effective care navigator seemed to overcome other potential barriers to care, such as where participants lived. However, if services where geographically outside the care navigators reach, they were unable to link people with dementia and carers to these services, which created a structural barrier by only a few kilometres of geographical placement and residency. This was particularly noted in the Netherlands, with limited reports of a care navigator in the UK, showing a lack of a particular type of support or link in the country.

*“Our care navigator is like our helicopter who checks whether everything is fine. I don’t need to do that myself and that is, yes, very nice.” Dutch female carer, sister, age 47*.

*“It was not within the range of 25 km. That was the problem. The municipality wouldn’t reimburse the transportation costs because it was too far.” Dutch female carer, spouse, age 77*.

**Insecure funding of services**

Some carers had experienced sudden withdrawal of previously available services from their locality, leaving them without any support and leading to carers setting up their own support networks. These insecure funding streams, which only fund some services for a short length of time, are noticeable structural barriers in accessing care and can cause reluctance in accessing services knowing funding could be withdrawn at any time.

*“we did start off with them quite well, they did a lot of support initially that was really good and then they pulled the plug out and just left everybody high and dry.” UK Female carer, spouse, ID08*.

**Diagnosis and information overload at first**

Receiving a diagnosis was considered to be the key to receiving support for the dementia. Some participants in the Netherlands felt more positive about the services they received, and felt fully supported, due to the diagnosis. However, this is not usually the case in the UK. Whilst a diagnosis is equally considered a vital step towards receiving support, carers were not always receiving all the support they or their relative required, and getting a diagnosis in the first place can often be difficult.

*“We have a diagnosis. We have all the help we need. And (pwd) is happy again. Despite… receiving such a diagnosis. (…) But ever since she officially received the diagnosis, many doors have opened. So that is just… just really nice.” Dutch Female carer, sister, age 47*.

*“We […] had to have her checked at the hospital and in checking her at the hospital a brain scan was done and that’s how we got diagnosis. I guess in a way all it did was ratify what we already knew.” UK Female carer, daughter, ID06*.

*“Each time, I had to manage things myself. […] it took more than a year before eventually with that doctor, that I did something about it. Before I finally got diagnosed, I think it took 1,5 to 2 years.” Dutch person with dementia*.

Receiving information about the dementia and caring aspects, as well as about services to contact, is one form of receiving support. However, carers often complained about receiving too much information at first at the point of diagnosis, when they are already overwhelmed emotionally with receiving and dealing with the diagnosis, yet experiencing a lack of information throughout the dementia. Instead, carers would benefit from having continued support throughout the diagnosis of their relative, including specific information which may be more suitable at a later stage, such as changes which may occur in the dementia in the more advanced stages. Receiving such information all together results in an information overload, leaving carers little room to process.

*“I think there was too much information, it was overwhelming, a lot of it people, it was a good start, I mean it was a start anyway erm but then when you come home and you start to deal with things as they occur you’re again overwhelmed.” UK ID03, male carer, husband*.

**THEME 5: Financing care**

The issue of financing dementia care was brought up as both a personal, and at times structural, barrier to accessing care. Some services are provided for free or are subsidised by the local authority or council, for example for paid home care or carers allowance in the UK. However, this usually does not suffice and most carers and people living with dementia have to pay for additional services. The issue of financing care can be particularly pronounced surrounding care home residency, which is the most cost-heavy element of care. Understanding who is eligible for support and how to access financial support was not always clear though, and could act as a barrier.

*“my Mum was spent all her limited savings just in those few months at the care home, so she was eligible for Council support. So I went down to the [location] offices and they seemed to perform some very whizzy calculations and decided my Mum would be eligible, not for housing support but for the money paid for the care. So we had to set up a separate account so they were great, the instructions were a bit unclear which did cause some problem later on about the finance.” UK Male carer, son, ID01*.

In the Netherlands, most care provided is reimbursed through health insurance or covered by the Social Support Act (WMO) through the municipalities; however, there can be bureaucratic barriers which hinder receiving care, e.g., limited number of sessions at a psychologist being compensated, or no compensation for day care too far from home. However, when the services are outside a certain geographical range, these will not be reimbursed for the person with dementia or carer, creating a financial barrier to accessing the care if they lack the funds to pay for the service themselves.

*“It was not within the range of 25 km. That was the problem. The municipality wouldn’t reimburse the transportation costs because it was too far.” Dutch Female, spouse, age 77*.

## 4. Discussion

This is one of the first studies which compares the barriers to accessing post-diagnostic dementia care between countries and cultural settings. People living with dementia and informal carers in the UK and the Netherlands experienced a number of personal and structural barriers to accessing post-diagnostic dementia care, at a time point shortly before the World Health Organisation announced COVID-19 as a pandemic outbreak. In particular, service structures seemed to slightly vary between the UK and the Netherlands, with one link key worker provided offering increased access to post-diagnostic support in the Netherlands, whilst similarities including a lack of person-centredness of many of the care services provided and thus often deemed unsuitable to the person with dementia, or the informal carer.

People living with dementia and informal carers accessed different types of social support services, ranging from day care centres, peer support groups, befrienders, and paid home care. Most people only utilised one service, and the most frequently used services were support groups and equipment. Accessing these services was at times fully or partially self-funded by carers or their relatives with dementia. Considering that services are provided by different outlets, including health care services, local councils and authorities, as well as third sector organisations, each service is likely having different forms of access in terms of free and paid care provision. This reliance on self-funding some services can be an inequity to dementia care, with more affluent people being more likely to access the care they need. Whilst most of these services are social services, such as day care centres and paid home care, as opposed to psychological support or healthcare access, this potential inequity is aligned with Tudor Hart’s Inverse Care Law [33], where more affluent people are in less need of access to medical care due to better living conditions, yet access the largest amount of care. In reverse, those from more socio-economically disadvantaged backgrounds are in greatest need of medical care, but access the least. A great deal of research has focused on accessing medical care in general, but financing issues in dementia care are starting to emerge [24], which is advanced by both the survey and interview results from our study. The financial side of utilising dementia care can thus be both a personal barrier, due to potentially limited financial means, but also a structural barrier to care, by services not being subsidised by the government to enable equitable access.

Even if people with dementia and carers may have the financial means however to access care, a prerequisite is to know about the services in the first place and accepting care. Knowledge about existing services is often unfortunately not always available, with carers reporting a severe lack of awareness of services and the need to be proactive to finding out about services. This is supporting recent evidence from England, exploring qualitatively specifically the barriers in accessing dementia care encountered in those living with and caring for someone with young-onset and late-onset dementia [12]. Moreover, there appears to be a general lack of understanding about dementia in carers, which can act as a barrier to seeking out help also [34], as carers may not associate certain symptoms with the dementia, but consider them to be part of the person’s character, and thus fail to seek support. Thus, there needs to be a greater shift towards raising awareness of available services and how to access them, but also the very basics of dementia and informing people about the different symptoms. Even when knowledge about services exists, people with dementia and carers also need to accept the care on offer to utilise the services. Boots and colleagues [35] reported how carers in particular can experience difficulties in acknowledging their needs, as they are concerned over stigma, thus impacting on their willingness to accept care. To overcome this personal barrier, early therapeutic interventions to provide information and address the issue of stigma and accepting help can therefore be helpful in increasing uptake of services.

One way in which access could be better enabled would be via a care navigator or link person. This seemed to be happening in the Netherlands, and highlighted the benefits of the person. In contrast, UK participants expressed a desire for one regular point of contact, as they felt lost within the system once a diagnosis was made. Some had experienced the benefits of an Admiral Nurse, which is a specialist dementia nurse of which there are only few across the country. Admiral Nurses are generally found to be a helpful link person in navigating the difficult terrain of post diagnosis [36,37], yet unfortunately there are too few nurses across the UK to cover all 920,000 people with dementia [2]. The need and benefits of a single point of contact after the diagnosis has also been highlighted in a recent European-wide study exploring access to formal dementia care [38,39] as well as in online carer blogs written about dementia and the end of life care stage [40]. Kerpershoek and colleagues also highlighted a number of other fixed and personal factors enabling access to formal dementia care across the eight European countries, including lack of information and attitudes towards accepting care. Whilst the authors explored these issues qualitatively across a larger country sample, our research supports these findings on accessing dementia care both quantitatively and qualitatively, whilst also providing a unique insight shortly before the pandemic outbreak, thus providing a baseline of inequalities up until dementia care has inevitably changed.

The pandemic has thrown up further barriers to accessing dementia care, whilst exacerbating existing ones [12,26]. Social support services suddenly stopped operating due to social distancing restrictions and lockdown to stem the spread of the virus, leaving many people unsupported and emotionally overwhelmed, including informal carers picking up additional caring duties resulting in increased levels of burden [41]. This is further amplified by difficulties in accessing remote services for people with dementia, where these services are and can be provided digitally, due to digital illiteracy and condition-specific difficulties [26]. This has not been found in the present study, as there was no need for remote care and people were only discussing their experiences of face-to-face services. An additional novel personal barrier which the pandemic seems to have created is a reluctance by people with dementia to re-emerge after lockdowns for fear of being able to engage in everyday tasks and meetings [27]. This is likely going to impact on their desire to access services, and with limited digital literacy, this may leave a large proportion of people with dementia without vital social support living in the community.

Whilst this study benefits from a mixed-methods approach and cross-country data, there are some limitations to consider. The Dutch questionnaire sample was smaller than the English sample, which was due to delays in starting recruitment as well as COVID-19 suddenly stopping recruitment in both countries all together. Thus, questionnaire data were more representative of the English carer population, highlighting a need for future research to conduct a larger study with equal sample sizes. Furthermore, there was a limited uptake of either the questionnaire or interview from carers from ethnic minority groups, which further limits the representativeness of the study’s findings. This is particularly important as people with dementia and carers from minority ethnic backgrounds experience specific barriers in accessing care, which have not been covered in this study [42,43]. In addition, the sample itself may be biased and lacks a degree of representativeness considering that participants were all recruited via memory services and support groups, thus participants had already accessed services. However, some have only received a diagnosis via their memory service, and have not accessed any subsequent services. Lastly, two different interviewers conducted the interviews (CG in the UK and AB in the Netherlands). Although both were fully trained in conducting qualitative research, and the same topic guide was employed, there may have been some minor variations in how interviews were conducted.

## 5. Conclusions

This study adds to a growing body of literature on the many barriers which people living with dementia and informal carers are facing when accessing formal dementia care. Whilst COVID-19 has changed how many care services are accessed, this study provides recommendations for facilitating access to care, such as having one link person or care navigator providing continued support throughout the dementia trajectory, as well as raising awareness of available services. Tackling the financial barriers to using services will rely on commissioning bodies and social care funding from governments, and policy guidance can be made based on those findings. More research needs to be conducted in the long-term throughout the pandemic and post-pandemic, however, on a wider international scale, to fully understand how inequalities in care access can be addressed, including in lower- and middle-income countries which face very different service provision.

## Figures and Tables

**Table 1:** Participant characteristics.

	Survey (*n* = 103)
**Person with Dementia**	
Age, Mean (SD)	78 (±8)
Years of education, Mean (SD)	12 (±3)
Years since diagnosis, Mean (SD)	4 (±7)
**N (%)**	
Gender	
Female	74 (71.8%)
Male	29 (28.2%)
Type of dementia	
Alzheimer’s disease	58 (57.4%)
Vascular dementia	21 (20.8%)
Other dementias	24 (21.8%)
Living situation	
Alone	15 (14.6%)
With family/friends	71 (68.9%)
Care home	17 (16.5%)
Country	
England	89 (86.4%)
Netherlands	14 (13.6%)
**Informal carer**	
Age, Mean (SD)	67 (±10)
Years of education, Mean (SD)	14 (±4)
**N (%)**	
Gender	
Female	54 (52.4%)
Male	49 (47.6%)
Relationship with PLWD	
Spouse/partner	52 (59.1%)
Adult child/in-law child	31 (35.2%)
Other	5 (5.7%)

**Table 2:** Service usage.

Service	N (%)	Self-Funded Fully or Partially (N (%))
Paid carers	30 (29.1)	16 (57.1)
PLWD Support groups	54 (52.4)	8 (21.6)
Carer support groups	31 (30.1)	6 (31.6)
Respite care	11 (10.7)	7 (63.7)
Day care centres	22 (21.4)	11 (52.3)
Care home	19 (18.4)	11 (57.9)
Home meals	9 (8.7)	4 (80.0)
Transport	18 (17.5)	3 (30.0)
Befriending	9 (8.7)	2 (33.3)
Clinical support	29 (28.2)	0
Equipment	37 (35.9)	9 (29.0)
Other	12 (11.7)	4 (40.0)

Legend. PLWD = Person living with dementia. Equipment includes any assistive technology and modification to the home environment, such as hand rails, specific clocks, medication dispensers, etc. The percentage of fully/partially self-funded cases includes some missing data. Whilst participants have ticked whether they receive certain types of services, some have left the funding information empty, which is why the percentage is based on those who have provided data.

**Table 3:** List of themes and subthemes.

Themes	Sub-Themes
Health literacy	Knowledge and communication skills A need to be proactive
Accepting help	
Service suitability	
Structural service barriers and enablers	Having one link person Insecure funding of services Diagnosis and information overload at first
Financing care	

## Data Availability

Anonymised data may be available upon reasonable request, but would need to be confirmed via the ethics board.

## Appendix A. Interview Guide

1. What types of services to support you with your dementia/ to support you caring for your relative with dementia have you accessed in the past and are you using at the moment?
  → Which basis was used to decide upon the currently/ past used services you use? (E.g. dementia-case-manager previously assessed living situation).
  → Who offered these services to you? Alternatively, where did you receive that information?
2. Do you have to pay to access these services yourself, and/or do you get financial support? If so, from which organisation?
  → If you receive financial support, how did you attain that support?
3. What are your experiences of accessing these services?
4. Have these services helped you?
  If not: was there support to better-fitted care? Who provided that support to you?
5. Do you need more support services to help you in your day-to-day life/ help you as a carer? Are there any barriers for you to access these?
6. When deciding upon suitable help, was there a jointed decision-making process in your opinion? (E.g. Is the PwD / other family members and/or a formal caregiver actively involved in the process?) How?
7. Where there alternative services offered to you as well?
  → Were they fitting your current living situation? Why not?
  → If fitting: which made you decide upon not taking these services?
